# Possible Implication of Fc****γ**** Receptor-Mediated Trogocytosis in Susceptibility to Systemic Autoimmune Disease

**DOI:** 10.1155/2013/345745

**Published:** 2013-09-04

**Authors:** Sakiko Masuda, Sari Iwasaki, Utano Tomaru, Tomohisa Baba, Kazuaki Katsumata, Akihiro Ishizu

**Affiliations:** ^1^Graduate School of Health Sciences, Hokkaido University, Sapporo 060-0812, Japan; ^2^Faculty of Health Sciences, Hokkaido University, Sapporo 060-0812, Japan; ^3^Department of Pathology, Hokkaido University Graduate School of Medicine, Sapporo 060-8638, Japan; ^4^Division of Molecular Bioregulation, Cancer Research Institute, Kanazawa University, Kanazawa 920-1192, Japan

## Abstract

Leukocytes can “gnaw away” the plasma membrane of other cells. This phenomenon, called trogocytosis, occurs subsequent to cell-to-cell adhesion. Currently, two mechanisms of trogocytosis, adhesion molecule-mediated trogocytosis and Fc**γ** receptor-(Fc**γ**R-) mediated trogocytosis, have been identified. In our earlier study, we established an *in vitro* model of Fc**γ**R-mediated trogocytosis, namely, CD8 translocation model from T cells to neutrophils. By using this model, we demonstrated that the molecules transferred to neutrophils via Fc**γ**R-mediated trogocytosis were taken into the cytoplasm immediately. This result suggests that the chance of molecules transferred via Fc**γ**R-mediated trogocytosis to play a role on the cell surface could be time-limited. Thus, we consider the physiological role of Fc**γ**R-mediated trogocytosis as a means to remove antibodies (Abs) that bind with self-molecules rather than to extract molecules from other cells. This concept means that Fc**γ**R-mediated trogocytosis can be a defense mechanism to Ab-mediated autoimmune response. Moreover, the activity of Fc**γ**R-mediated trogocytosis was revealed to be parallel to the endocytotic activity of neutrophils, which was critically related to the susceptibility to systemic autoimmune diseases. The collective findings suggest that Fc**γ**R-mediated trogocytosis could physiologically play a role in removal of Abs bound to self-antigens and prevent autoimmune diseases.

## 1. Introduction

Trogocytosis is the exchange of plasma membrane fragments between immune cells which form a conjugate [1, 2]. The phenomenon was first described on CD8^+^ T cells [3]. The CD8^+^ T cells can take plasma membrane fragments of antigen-presenting cells (APCs) via T-cell receptor (TCR) and antigen (Ag)/class I major histocompatibility complex (MHC), when these cells form an immunological synapse. To date, it has been shown that not only CD8^+^ T cells but also other immune cells, including CD4^+^ T cells [4], *γδ*T cells [5], B cells [6], natural killer cells [7], dendritic cells [8], monocytes [9], and macrophages [10], have potential for trogocytosis. These cells are able to accept plasma membrane fragments of other cells after recognition of cell surface Ags by the specific receptors. This mechanism is called adhesion molecule-mediated trogocytosis. 

Recently, another mechanism of trogocytosis mediated by Ag/antibody (Ab) immune complex and Fc*γ* receptor (Fc*γ*R), namely, Fc*γ*R-mediated trogocytosis, is advocated [11–13]. The major difference of Fc*γ*R-mediated trogocytosis from adhesion molecule-mediated trogocytosis is the intervention of Ab. This phenomenon is well characterized, wherein CD20 molecules on malignant B cells are lost after infusion of the humanized anti-CD20 monoclonal Ab, rituximab [14–16]. In this situation, Fc*γ*R^+^ immune cells capture the plasma membrane fragments of B cells via the CD20/anti-CD20 immune complexes and Fc*γ*Rs. Similar phenomenon is seen when CD22 [17] and CD25 [18] are targeted by therapeutic Abs. Although the best known cells that can perform Fc*γ*R-mediated trogocytosis are monocytes and macrophages [12, 19–21], the most abundant Fc*γ*R^+^ cells in peripheral blood, neutrophils, also have potential for Fc*γ*R-mediated trogocytosis [22, 23].

The physiological roles of trogocytosis have been discussed concerning T cells that acquired the Ag/MHC from the APCs in particular [24, 25]. The acceptor CD8^+^ T cells for the Ag/MHC are prone to be killed by the fratricide mechanism. In another way, the acquisition of HLA-G1 from APCs via trogocytosis could change the T-cell phenotype from effector to regulatory phenotype [26, 27]. It has been also demonstrated that CD4 CD8 double negative regulatory T cells, which accept the Ag/MHC from APCs via trogocytosis, can function as cytotoxic cells toward the Ag-specific CD8^+^ T cells [28]. These could be related to the convergence or suppression of the immune response. On the contrary, it is indicated that the T cells with the Ag/MHC can possibly function as new APCs resulting in the progression of the immune response. These controversial properties of T cells through adhesion molecule-mediated trogocytosis could be displayed in time and environment-dependent manners. In addition, recent studies have demonstrated that adhesion molecule-mediated trogocytosis could be related to the exercise of naturally occurring FOXP3^+^ regulatory T cells [29]. However, the physiological role of Fc*γ*R-mediated trogocytosis remains unrevealed. In this perspective, the role of Fc*γ*R-mediated trogocytosis in the physiological immune system is discussed according to the current data.

## 2. Establishment of *In Vitro* Model of Fc**γ**R-Mediated Trogocytosis

**Figure 1:**
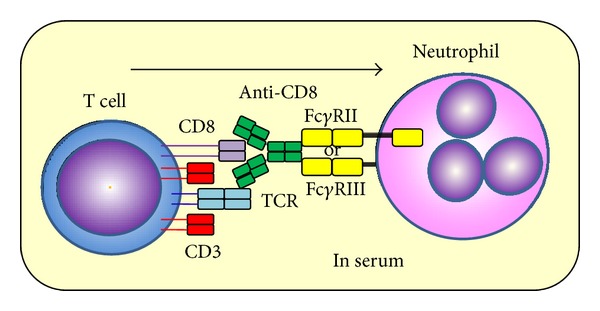
Schemas of CD8 translocation model from T cells to neutrophils CD8 molecules on T cells were transferred to neutrophils when these cells were incubated with anti-CD8 Ab in the serum. TCR and CD3 molecules on T cells were also transferred to neutrophils. Fc*γ*Rs (Fc*γ*RII and Fc*γ*RIII) were involved in the mechanism.

Daubeuf et al. had established a simple method to detect adhesion molecule-mediated trogocytosis by flow cytometry (FCM) [30]. On the other hand, we earlier established an *in vitro* model of Fc*γ*R-mediated trogocytosis [23]. The protocol was simple and easy as follows: (1) heparinized peripheral blood samples were incubated with anti-CD8 and anti-CD15 Abs: (2) after removal of erythrocytes by treatment with ammonium chloride, cells were subjected to FCM. Results demonstrated the presence of CD8^+^ cells in CD15^+^ neutrophils. Since neutrophils do not express CD8 innately, the CD8 molecules detected on neutrophils seem to be derived from other cells. Our studies revealed that CD8 molecules on T cells were transferred to neutrophils via the anti-CD8 Ab and Fc*γ*Rs (Fc*γ*RII and Fc*γ*RIII) on neutrophils. The usage of Fc*γ*RIII appears to be a specific characteristic of neutrophils because Fc*γ*RII, especially Fc*γ*RIIB, among the Fc*γ*Rs mediates trogocytosis in monocytes and macrophages [19, 31]. Moreover, bystander molecules, such as TCR and CD3, were also transferred from T cells to neutrophils accompanied by CD8 molecules. Thus, this phenomenon was considered as Fc*γ*R-mediated trogocytosis (Figure 1). The polymerization of actin was involved in the process of neutrophil Fc*γ*R-mediated trogocytosis similar to T cells [32]. By using this model, we demonstrated that human anti-mouse IgG Abs in serum accelerated Fc*γ*R-mediated trogocytosis. This is consistent with the finding that Fc*γ*R-mediated trogocytosis is prone to occur in arthritic patients positive for rheumatoid factor, which is an anti-IgG Ab [33].

## 3. Immediate Intake of Molecules Deprived via Fc**γ**R-Mediated Trogocytosis

**Figure 2:**
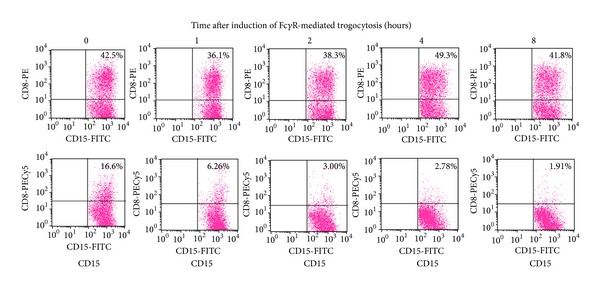
Dynamism of molecules transferred to neutrophils via Fc*γ*R-mediated trogocytosis. Heparinized peripheral blood samples (100 *μ*L) were made to react with 0.1 *μ*g/mL of PE-labeled anti-CD8 Ab for 30 min at room temperature. PE-labeled mouse IgG1 was used as an isotype-matched control. After depletion of erythrocytes, the cells were incubated in RPMI-1640 medium with 10% FBS for 0–8 hours at 37°C. Subsequently, the cells were resuspended in 100 *μ*L of PBS and then made to react with 0.1 *μ*g of PECy5-labeled anti-CD8 Ab for 20 min at room temperature. After removal of unbounded Ab, the cells were resuspended in 100 *μ*L of PBS followed by reaction with 0.1 *μ*g of FITC-labeled anti-CD15 Ab for 20 min at room temperature and then served for FCM. The PECy5-labeled anti-CD8 Ab used in this experiment could recognize a different epitope from that recognized by the PE-labeled anti-CD8 Ab. Experiments were repeated at least 3 times. Similar results were reproduced.

By using the *in vitro* model of Fc*γ*R-mediated trogocytosis, namely, CD8 translocation model from T cells to neutrophils, the dynamism of the molecules deprived by Fc*γ*R^+^ cells via Fc*γ*R-mediated trogocytosis was determined. For this purpose, two anti-CD8 monoclonal Abs that could recognize diverse epitopes were applied. First, CD8 molecules on T cells were made to transfer to neutrophils via Fc*γ*R-mediated trogocytosis using the PE-labeled anti-CD8 Ab. After incubation for 0–8 hours, CD8 molecules that remained on the cell surface of neutrophils were detected by the other PECy5-labeled anti-CD8 Ab. Although the number of neutrophils labeled by PE (neutrophils that performed Fc*γ*R-mediated trogocytosis) was substantially stable during the incubation period, the number of PECy5-labeled CD8 molecules on the cell surface of neutrophils diminished and reached low level by 2 hours (Figure 2). These findings indicated that the molecules transferred to neutrophils via Fc*γ*R-mediated trogocytosis were taken into the cytoplasm immediately.

## 4. Association between Fc**γ**R-Mediated Trogocytosis and Endocytosis 

**Figure 3:**
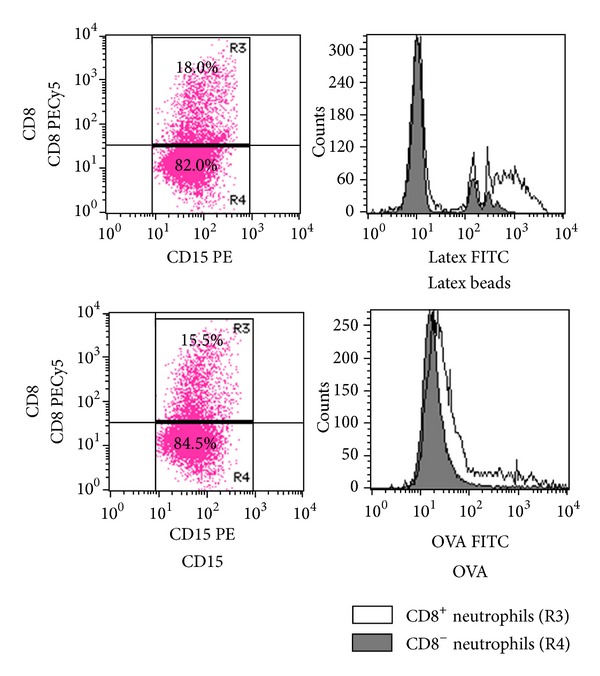
Comparison of endocytotic activities between neutrophils with and without display of Fc*γ*R-medicated trogocytosis. Peripheral blood polymorphonuclear cells (0.5 × 10^6^) and mononuclear cells (0.5 × 10^6^) were mixed and preincubated in 100 *μ*L of the autologous serum for 20 min at 37°C. The cells were made to react with 0.1 *μ*g of PECy5-labeled anti-CD8 Ab for 1 hour at room temperature. After washing with PBS, the cells were resuspended in 1 mL of RPMI-1640 medium with 10% FBS. Subsequently, the cells were made to react with 2 *μ*L of yellow-green carboxylate-modified latex beads for 90 min at 37°C or with 100 *μ*g of FITC-labeled OVA for 30 min at 37°C. After washing 3 times with PBS, the cells were re-suspended in 100 *μ*L of PBS and then made to react with 0.1 *μ*g of PE-labeled anti-CD15 Ab, followed by serving for FCM. Experiments were repeated at least 3 times. Similar results were reproduced.

Next, the association between Fc*γ*R-mediated trogocytosis and endocytosis was examined using the CD8 translocation model from T cells to neutrophils. Endocytosis is a fundamental function of neutrophils. Two diverse mechanisms of endocytosis include phagocytosis and pinocytosis, wherein the difference between the two is the size of target molecules. Pinocytosis is used for the absorption of extracellular fluids, and in contrast to phagocytosis, the target molecules are very small. To determine the association between Fc*γ*R-mediated trogocytosis and endocytosis, experiments were performed using yellow-green carboxylate-modified latex beads and FITC-labeled ovalbumin (OVA). The process of intake of latex bead and OVA represents phagocytosis and pinocytosis, respectively. After enhancing Fc*γ*R-mediated trogocytosis by the CD8 translocation model from T cells to neutrophils, fluorescence-labeled latex beads and OVA were added to the cells. Subsequently, the engulfment of these molecules by CD8^+^ neutrophils that underwent Fc*γ*R-mediated trogocytosis was examined, and the data were compared with those by CD8^−^ neutrophils that did not perform Fc*γ*R-mediated trogocytosis. The activities of both phagocytosis and pinocytosis were higher in CD8^+^ neutrophils than those in CD8^−^ neutrophils (Figure 3). These findings suggest that the activity of Fc*γ*R-mediated trogocytosis is parallel to the phagocytic and pinocytic activities of neutrophils.

## 5. Physiological Role of Fc**γ**R-Mediated Trogocytosis

**Figure 4:**
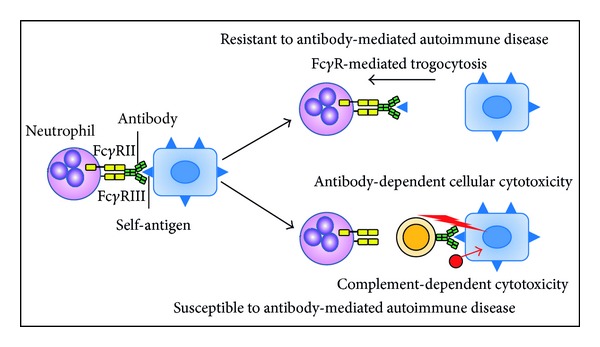
Relation between Fc*γ*R-mediated trogocytosis and susceptibility to Ab-mediated autoimmune disease. Fc*γ*R-mediated trogocytosis can play a role in the removal of Abs that bind to self-Ags on the cell surface and prevent Ab-mediated autoimmune diseases.

It was revealed that the CD8 molecules transferred to neutrophils via Fc*γ*R-mediated trogocytosis were taken into the cytoplasm immediately. This evidence suggests that the chance of molecules transferred via Fc*γ*R-mediated trogocytosis to play a role on the cell surface could be time-limited. Thus, we consider the physiological role of Fc*γ*R-mediated trogocytosis as a means to remove Abs that bind with self-molecules rather than to extract molecules from other cells. Essentially, a living body is always exposed to invading pathogens. The immune system produces Abs to compete with the pathogens; however, these Abs sometimes cross-react with self-Ags. In such situation, the presence of mechanism that can prevent Ab-mediated autoimmune response is beneficial. Fc*γ*R-mediated trogocytosis can be a defense mechanism to remove Abs, which unexpectedly bind to self-Ags (Figure 4).

In this study, the relationship between Fc*γ*R-mediated trogocytosis and endocytosis of neutrophils was examined using the established CD8 translocation model from T cells to neutrophils. Results clearly indicated that the activity of Fc*γ*R-mediated trogocytosis was closely linked to the endocytosis activity of neutrophils. The low activity of endocytosis of neutrophils is critically related to the high susceptibility to systemic autoimmune diseases including systemic lupus erythematosus [34]. Therefore, it is reasonable to consider that the low ability of Fc*γ*R-mediated trogocytosis could be also linked to the high susceptibility to systemic autoimmune diseases. This is compatible with our concept that Fc*γ*R-mediated trogocytosis can play a role in the removal of autoantibodies and prevent autoimmune diseases. However, in autoimmune hemolytic anemia, trogocytosis mediated by anti-red blood cell (RBC) polymeric IgA Abs and Fc*α* receptors is involved in the destruction of RBCs [35]. Thus, further prospective studies are needed to clarify if the ability of Fc*γ*R-mediated trogocytosis would be actually related to the susceptibility to systemic autoimmune diseases.
